# Hypersensitivity Reactions in Serious Adverse Events Reported for Paracetamol in the EudraVigilance Database, 2007–2018

**DOI:** 10.3390/pharmacy7010012

**Published:** 2019-01-17

**Authors:** Iwona Popiołek, Katarzyna Piotrowicz-Wójcik, Grzegorz Porebski

**Affiliations:** 1 Toxicology Ward, University Hospital in Krakow, Sniadeckich 10, 31-531 Krakow, Poland; gawlikiwona2.0@gmail.com; 2 Department of Clinical and Environmental Allergology, Jagiellonian University Medical College, 31-531 Krakow, Poland; katarzyna.piotrowicz-wojcik@uj.edu.pl

**Keywords:** paracetamol, drug hypersensitivity reaction, over-the-counter drugs, adverse events, drug safety, spontaneous reporting

## Abstract

Paracetamol is a popular and easily available drug which is used world-wide as analgesic, antipyretic agent. Hypersensitivity reactions to this drug involve a wide range of symptoms of various importance for patient management. The EudraVigilance (EV) database serves as a system for monitoring adverse events (AE) due to drug intake. We retrospectively recorded AE reports for “paracetamol” reported from 1 January 2007 to 1 October 2018 which fulfilled the category of “serious” in EV. For further analysis the retrieved AE reports were selected according to the keywords corresponding to hypersensitivity symptoms. We included in the study 4589 AE reports with 9489 particular AEs. 24.2% of all the AE reports concerned children. The most often reported symptoms were “angioedema,” “rash” and “urticaria” (each of them with a frequency of >10% in the AE reports). An important group of AEs were oedema reported as being located in the head, neck or respiratory tract. We recorded 58 AE reports with fatal outcomes, including 9 Stevens-Johnson syndrome/toxic epidermal necrolysis cases (SJS/TEN), 10 anaphylactic reactions, 21 cases of hepatic failure and a further 18 cases which occurred for other reasons. SJS/TEN, acute generalized exanthematous pustulosis and drug reaction with eosinophilia and systemic symptoms were reported 129, 42 and 25 times, respectively. Prodromes and symptoms of potentially life-threating SJS/TEN appeared in 286 of the AE reports. 380 AE reports pointed to a diagnosis of anaphylaxis. To improve patient safety, healthcare professionals, including pharmacists, can identify warning signs of severe hypersensitivity reactions to paracetamol.

## 1. Introduction

Paracetamol (acetaminophen, Anatomical Therapeutic Chemical Classification code N02BE01) is one of the most popular over-the-counter (OTC) drug used worldwide and available without a prescription [1,2,3]. Despite its relative safety, a significant number of adverse event (AE) reports is annually collected by European Medicines Agency (EMA). The most common reported symptoms were nausea and vomiting, followed by increased aspartate aminotransferase, abdominal pain, hypersensitivity reactions, haemorrhages, anaemia, rash, pruritus, dyspnoea, peripheral oedema, headache, dizziness, muscle spasms, insomnia and others [4]. It is important for pharmacists to be aware of adverse events, both the more frequent ones and also those that occur more rarely but which are more serious in their outcomes. Patients who use this medicine without prescription get information about this medicine from advertisements. Pharmacists are sometimes the only professionals who may warn patients about safety issues. Data collected in Europe revealed increased mortality in population after non-intentional overdose [5].

EudraVigilance (EV), the European database for adverse events reports, is the tool that the European Medicines Agency (EMA) and the national competent authorities use for monitoring the safety of all authorised medicines on the European Union (EU) market [6]. EV is a public access system. In Directive of the European Commission (EC) 2001/83/EC, an adverse reaction was defined as a response to a medicinal product which is noxious and unintended [7]. In 2012, the definition was extended to all symptoms which can be related to drug in general—including overdose—and the nomenclature was also changed to adverse event, which corresponds to any harmful effect of drug intake. Spontaneous reporting collected in EV is triggered by the suspicion of a healthcare professional or a patient who has observed signs and symptoms which could have been caused by a medicine. EudraVigilance in 2017 keeps information on more than 12.45 million safety reports which refer to 7.95 million cases, as well as keeping information on 744,219 of the medicinal products which are available on the EU market [8].

Allergists distinguish type A and type B of adverse drug reactions (ADR) [9]. Type A is usually related to the size of the dose. This reaction is the consequence of the pharmacological mechanism of the drug. Type B reactions are not dose-dependent, these reactions are mostly hypersensitivity reactions to the drug [9,10]. For paracetamol, the most important type A ADR is the hepatotoxic effect of the N-acetyl-p-benzoquinone imine (NAPQI) non-primary, cytochrome pathway metabolism-product [11]. Type B ADR, hypersensitivity may be presented by numerous symptoms, mainly by the presence of localised oedema and are related to skin and mucosa [12]. Although adverse reactions to paracetamol are rare, at times they can cause life-threatening conditions [13]. Stevens-Johnson syndrome (SJS) is one of such potentially lethal adverse drug reaction. Most of the reported cases of analgesic-induced SJS were due to oxicams or propionic acid derivatives [13]. In 2013, the Food and Drug Administration (FDA) announced that acetaminophen can cause serious or even potentially fatal skin reactions such as acute generalized exanthematous pustulosis (AGEP) and can also cause Stevens-Johnson syndrome (SJS), which is called toxic epidermal necrolysis (TEN) when the skin lesions cover more than 30% of the patient’s body surface [14].

A significant part of adverse event reports are related to overdoses of paracetamol [15]. The symptoms are well-known to healthcare professionals working in emergency departments or toxicology wards. These cases are due to intentional poisoning by patients (suicide attempts) or the unintentional overdoses [15,16]. However type B ADR’s are a rare occurrence. Patients are not always aware of the consequences due to the large variability of possible reactions to paracetamol. Even for healthcare professionals it is challenging to establish a causal relationship between the exposure to a drug and the consequent hypersensitivity symptoms.

In this study, we have aimed to analyse spontaneous reporting on paracetamol hypersensitivity reactions in Eudra Vigilance database regarding: (i) trends which were observable during the period between 2008 and October 2018 (ii) the differences between healthcare and non-healthcare providers of spontaneous reports, (iii) the profile of reported symptoms and affected individuals and finally (iv) the most serious and life-threating reactions and their prodromes reported in EV, namely SJS, which is of the highest importance for drug safety both for the particular patient and for the healthcare system in general.

## 2. Materials and Methods

We retrospectively analysed AE reports for “paracetamol” reported from 1 January 2007 to 1 October 2018 publicly available in the EV database at www.adrreports.eu portal. To limit the results of our research to the most clinically relevant data, we included in the analysis only those AE reports which were termed as “serious” (defined in EV as: “adverse events which results in death,” those which are “life-threatening,” those which “require inpatient hospitalization or prolongation of existing hospitalization,” those which “results in persistent or significant disability or incapacity” or those which are the results of “a congenital anomaly/birth defect”) [7].

We analysed only the AE reports where paracetamol was the only reported drug of interest. AE reports with other concomitant medications and with paracetamol combined with other active substances were excluded from the analysis, as a single culprit drug cannot be pinpointed in such cases.

The extracted AE reports were searched according to keywords corresponding to hypersensitivity reactions (Supplementary Materials, Tables S1–S3) which were in their turn selected from the terms describing reactions in EV database. Keywords were chosen independently by a medical doctor who works with patients who suffer from adverse drug reactions and also by a trained allergist who specializes in research on drug hypersensitivity reactions. In cases of divergence, the keywords were designated upon agreement. The AE reports were assigned to “reaction groups,” categories utilized in the EV database (“skin and subcutaneous tissue disorders,” “eye disorders,” “general disorders and administration site conditions,” “vascular disorders,” “respiratory, thoracic and mediastinal disorders,” “immune system disorders,” “gastrointestinal disorders,” “reproductive system and breast disorders,” “ear and labyrinth disorders,” “investigations”). Age group, patient sex, outcome and information about overdose were included in the analysis. A search for prodromes or symptoms of SJS/TEN was performed with keywords marked with asterisk (*) in Tables S1–S3. Those keywords have been chosen arbitrary on the basis of existing knowledge and authors’ experience [17]. A search for the diagnosis of anaphylaxis was performed with keywords marked with a double asterisk (**) in Table S1.

## 3. Statistical analysis

Data were extracted from the EV database and transferred to large Microsoft Excel files and prepared for further investigation. The EV database contains categorical variables, namely: “Primary Source Qualification” (non-/healthcare professional), “Patient Age Group” (divided into five categories: 0–11 years old, 12–17 years old, 18–65 years old, more than 65 years old and unspecified), “Patient Sex,” “Reaction List” (including outcome and seriousness criteria), “Suspect Drug List,” “Concomitant Drug List.” Data have been presented by the means of descriptive statistics. Further statistical analysis was performed using *Statistica 13* software (TIBCO Software Inc., Palo Alto, CA, USA). Differences in proportions between the groups were compared with the Chi-square test. A P value of less than 0.05 was considered statistically significant.

## 4. Results

### Population Characteristic, Presentation of Results

**Figure 1:**
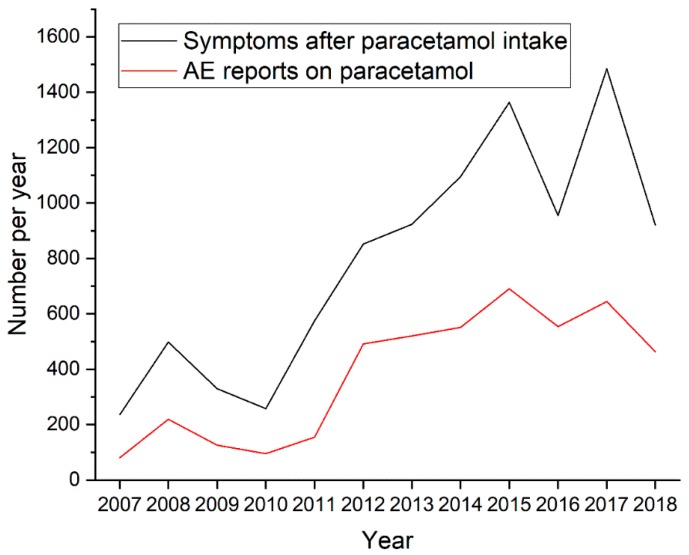
Time trends and comparison of number of symptoms and number of adverse events reports after paracetamol intake per year.

**Figure 2:**
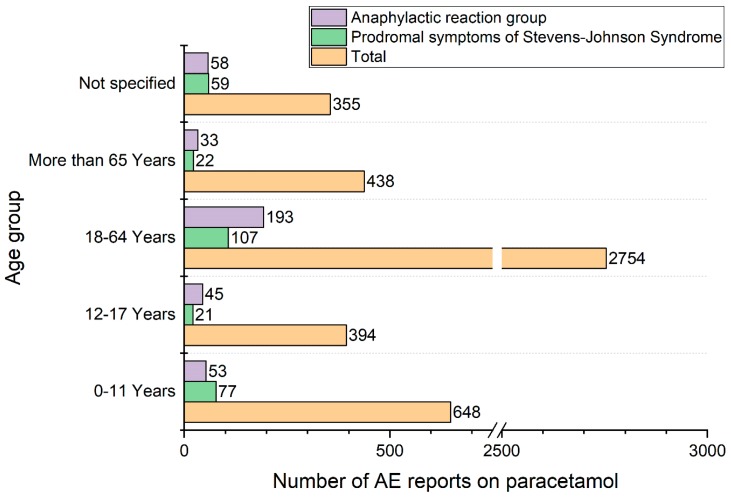
Age distribution in total and distinctively for symptoms related to the Stevens-Johnson syndrome and anaphylactic reactions after paracetamol intake.

In the studied period, the total number of serious AE reports for paracetamol was 16,810. Among them were 4589 serious AE reports related to hypersensitivity symptoms, 2653 (57.8%) of the subjects were female, 1813 (39.5%) of them were men and 123 (2.7%) reports did not specify the sex of those taking part. These reports contained a total of 9489 individual AEs; including 6288 Adverse Events related to states of hypersensitivity as described by the keywords presented in the Supplementary Materials Tables S1–S3. The mean number of AEs per report was 2.1 (range 1–33). As shown in Figure 1, the number of reports collected in the EV database increased over time. 1042 AE reports concerned children, including 648 cases of children below 12 years of age and 394 cases of teenagers from 12 to 17 years old. This accounts for 24.2% of all the AE reports in which data on age are specified. More detailed data regarding the age of the patients are presented in Figure 2.

**Figure 3:**
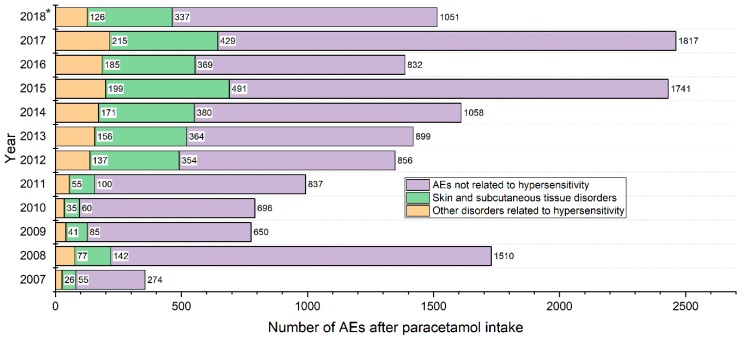
Time trends of the total number of Adverse Events (AE) reports, together with the number of skin and subcutaneous tissue disorder reports and other AE reports related to hypersensitivity. (*) Data for 2018 were collected from January to 1st October

The most frequent symptoms reported in analysed AEs were “angioedema,” “rash” and “urticaria.” All of them occurred with a frequency of above 10%. The proportion of female patients was slightly but significantly higher in the group of angioedema (60% vs 57%, p < 0.05) and of those with rash (59% vs 57%, p < 0.05), however there was no difference in sex distribution for urticaria. Furthermore, an important group of AEs were oedemas of the eye and orbital region (390 cases, 8.5%), as well as oedemas of the rest of the head and neck and also including oedemas of the respiratory system (319, 7.0%). Reports of oedema located elsewhere or with an unspecified location were less frequent. These symptoms were followed by anaphylactic signs and numerous skin manifestations of hypersensitivity, including the most severe ones (e.g. Stevens-Johnson syndrome), listed in detail in Table 1, presenting the most frequently occurring symptoms. The hypersensitivity symptoms reported as being an AE with a frequency <1.1% are shown in Supplementary Materials (Table S4). The results were organized according to “reaction groups,” categories utilized in the EV database and these results showed that the category “skin and subcutaneous tissue disorders” was most commonly involved in AE reports (Figure 3). It was followed by the categories: “eye disorders” (periorbital or orbital oedemas) and “immune system disorders.” The next most common categories with corresponding numbers of AEs are presented in Table 2.

**Figure 4:**
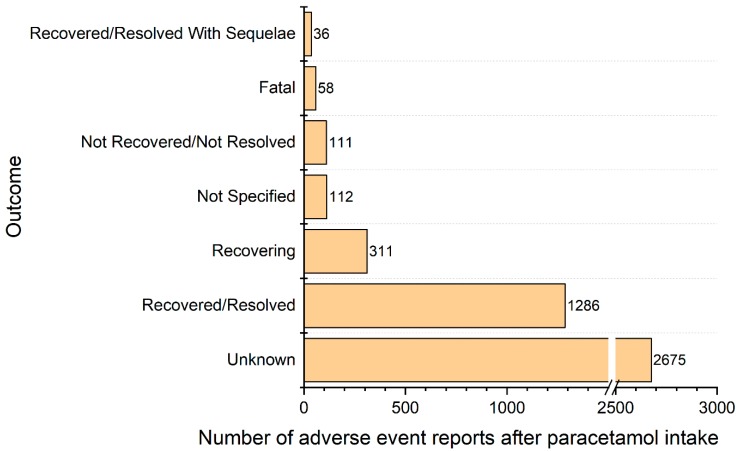
Outcomes reported after paracetamol-induced hypersensitivity reaction.

We recorded 58 AE reports with fatal outcomes (29 females, 26 males, 3 not specified sex), another 111 subjects did not recover, 36 recovered with sequelae and 1597 recovered completely or were reported as recovering. The further history of 58.3% subjects from the reports was unknown (Figure 4). In 2007–2011, the mean percentage of unknown outcomes was 16% and after 2012 rose to 66%. The unknown outcomes were more frequently recorded in non-healthcare professional reports than in the reports from healthcare professionals 69% versus 56%, respectively. The percentages of the unknown outcomes were distinct for particular symptoms, for example, anaphylactic reaction—29%, toxic epidermal necrolysis—32%, rash—61%, eye swelling—87%. Fatal cases were caused by: SJS/TEN (9), anaphylaxis (10), overdose accompanied by hypersensitivity (4), drug reaction with eosinophilia and systemic symptoms (3) and other reasons (32) including: hepatic failure with shock (15), hepatic failure without shock (6), respiratory tract symptoms (5), skin symptoms (4), coagulopathy (1), methemoglobinemia (1). Prodromes and symptoms of potentially life-threating SJS/TEN cases appeared in 286 AE reports (6.2% of all AE reports). Majority of those reports (244, 85.3%) included generalized reactions, namely: “Stevens-Johnson syndrome” (129), “toxic epidermal necrolysis” (108), “acute generalized exanthematous pustulosis" (2), “rash generalized” (2), “generalised erythema” (1), “drug reaction with eosinophilia and systemic symptoms” (1), “respiratory distress” (1). Those symptoms were found in all age groups (Figure 2) with a relative predominance in the youngest group (children <11 years old).

380 AE reports pointed to a diagnosis of anaphylaxis with the highest percentage (11.4%) in the age group (12–17 years old), as shown in Figure 2. Acute generalized exanthematous pustulosis was reported 42 times in 90.5% by professionals. We also recorded 25 cases of drug reactions with eosinophilia and systemic symptoms—96.0% of them were reported by healthcare professionals.

Healthcare professionals reported 82.5% (3786) of all AE cases, whereas non-healthcare professionals reported in only 17.0% of their cases. Comparison of AEs coming from healthcare providers and non-healthcare professionals revealed statistically significant differences in some symptoms, namely: “hypersensitivity,” “shock symptom” and “swelling” were reported more often by non-healthcare professionals, whereas healthcare providers reported more frequent “urticaria” and “toxic epidermal necrolysis.”

## 5. Discussion

In our analysis, more than one quarter (27.3%) of all serious AE reports connected to paracetamol were related to hypersensitivity reactions, therefore these reactions represent a significant clinical issue. Because of the fact that prescription data for over-the-counter drugs are difficult to obtain, the epidemiology of paracetamol hypersensitivity is poorly recognized [18]. Anaphylactic reactions to acetaminophen are thought to be immunologically or non-immunologically mediated [19,20]. Paracetamol functions as a weak inhibitor of cyclooxygenase-1 and it can induce reaction analogous to nonsteroidal anti-inflammatory drugs (NSAIDs) [20,21]. Cross-sensitivity between aspirin and acetaminophen in aspirin-sensitive asthmatic patients has been reported with frequencies ranging from 0–29% [21]. On the other hand, IgE-mediated acetaminophen-induced hypersensitivity is extremely rare [20]. The most common hypersensitivity reactions to analgesic are those caused by NSAIDs. The prevalence of hypersensitivity to NSAIDs has been estimated to be 0.5–1.9% of the general population. NSAIDs are responsible for 21–25% of all adverse reactions to drugs [22].

A moderate to strong predominance in females to react to paracetamol is a commonly observed phenomenon, described previously [23]. Female patients have a 1.5- to 1.7-fold greater risk of developing an ADR, including common adverse skin reactions (angioedema and rash), compared with male patients. The reason for this phenomenon is unknown. There are opinions that differences in circulating hormones may play a role or the lower body mass and water content also may increase the risk of adverse effect [23,24]. Also, an important factor may be a greater readiness to report adverse reactions [25,26].

The increasing number of reported AEs over time can be attributed to a better awareness of drug safety issues among healthcare professionals, who provided most of the analysed reports. However underreporting is common shortage of public healthcare databases based on spontaneous reporting [27]. Paracetamol was used in all investigated age groups (Figure 2), including children (0–11 years old) and adolescents (12–17 years old) who represented together 22.7% of the total number of AE reports. Paracetamol is the drug of choice for many medical needs in children [28], whereas adults may use a much wider range of pain-killer and antipyretic drugs, which include non-steroidal anti-inflammatory drugs. Nonetheless, the incidences of AEs are not an argument for abandoning paracetamol in favour of NSAIDs, since other analgesics or antipyretics are even less safe [29]. The extension of research into a comparison of the adverse reactions induced by paracetamol and by NSAIDs would be a valuable approach in future studies.

Paracetamol frequently causes skin hypersensitivity reactions as angioedema, urticaria, maculopapular exanthema and rash [30]. Many drugs can cause severe hypersensitivity symptoms like anaphylaxis, which is an acute, potentially fatal hypersensitivity reaction caused by the release of mediators from mast cells. The most common medication triggers are aspirin and parenterally given penicillin. There is a relatively low risk of anaphylaxis for paracetamol [31].

Stevens-Johnson syndrome/toxic epidermal necrolysis is an acute dermatosis characterized by epidermal loss and multisite mucositis, which is commonly accompanied by systemic disturbances. Numerous circular confluent lesions occur at the beginning on the upper torso, proximal limbs and face, thereafter lesions spread to the rest of the body. The skin is weak and the epidermis can easily peel back (Nikolsky’s sign) leaving areas of exposed dermis [32]. Involvement of the mucous membranes of the mouth, eyes or genitalia leads to exfoliation, erosion and ulceration which can cause serious haemorrhagic mucositis.

Acute generalized exanthematous pustulosis (AGEP), a serious skin reaction characterized by an acute onset of small non-follicular pustules on an erythematous base can be incidentally caused by acetaminophen. In about 20% of cases systemic involvement occurs [33]. Mortality is under 5% [34] and most patients improve within two weeks. Another serious drug reaction is DRESS (drug reaction with eosinophilia and systemic symptoms), whose pathogenesis is not fully understood but whose most probable mechanism is an immunological reaction to a drug or to drug metabolites. The main symptoms of this are rash, fever, lymphadenopathy, haematological abnormalities and systemic illness. The mortality rate for this reaction according to different studies ranges from 10 to 40% and depends on vital organ dysfunction [35]. In many cases an identification of the culprit drug is impossible or extremely difficult to make. In vivo tests in patients with serious life-threatening reactions are contraindicated. Fortunately laboratory diagnostics delivers some useful tools such as in vitro assays [36,37].

The completeness of data in EV depends on quality of particular AE reports. Moreover, privacy policy makes impossible an access to more detailed information on individual reports. The arbitrary choice of the keywords corresponding to prodromes of SJS is a different limitation of the study. Patients were more likely to describe medical conditions using less specialized vocabulary, for example using the word “swelling” rather than “angioedema.” This use of non-specialist vocabulary is a limitation of the study related to spontaneous reporting involving non-healthcare professionals. In addition, attention is paid to a large number of unknown outcomes in reports. Two main reason of unknown outcomes can be identified: (i) reporting by non-healthcare professionals (an overall higher percentage of unknown outcomes in this group and an increase in the number of unknown outcomes after 2012 when consumer reporting was accepted); (ii) relatively mild symptoms were followed by a higher percentage of unknown outcomes in comparison to severe symptoms (e.g. rash vs anaphylactic reaction). The necessity for providing the outcome is not a required piece of information for the acceptation the AE report in the EV database. The reports from non-healthcare professionals have clear drawbacks but patient reporting can still be useful in continuous monitoring of drug [38,39]. Another obvious limitation is the fact that collected data are very subjective and are not verified by additional diagnostic tests, which is important in the case of hypersensitivity symptoms. One should be also aware of the fact that the presented data cannot be used to determine the likelihood of experiencing a particular adverse event and that cases are reported on the basis of a suspicion of causal relationship between drug intake and adverse event, what does not necessarily mean that a link has been established. On the other hand, we believe that the strengths of the study are (i) the fact that it was a long investigation period (over a decade); (ii) the collection of data took place in real-life conditions which reflect common medical decisions of healthcare providers and/or patients; (iii) the presence of the identification of a reasonable number of AE reports on prodromes and symptoms of potentially life-threating medical conditions occurring after an intake of a widely-used drug which is known as a safety medication.

## 6. Conclusions

The role of pharmacists is crucial for detecting and warning patients about possible adverse events related to paracetamol. However most of the reported hypersensitivity symptoms are common (angioedema, rash, urticaria), due to the large the number of users some of the reported adverse events are far less typical. A reasonable number of patients experience some prodromes and symptoms of SJS/TEN, as well as other severe cutaneous skin reactions which can be followed by the increased risk of a fatal outcome. In addition, patients who take the OTC drug are focused on the symptoms of the disease that caused the need to take this medicine rather than on the new complaints which can be induced by drug. Therefore, they may easily miss the unexpected signs of paracetamol-induced hypersensitivity. Pharmacists can refer the patient to a physician at the early stage of hypersensitivity reaction for monitoring and further treatment.

## Figures and Tables

**Table 1:** Serious Adverse Events, attributed to paracetamol, reporting hypersensitivity symptoms and occurring with frequency >1.1% in EudraVigilance database (2007–2018). The total number of reports in the database is 4589, total number of Adverse Events described by the keywords presented in Supplementary Materials: Tables S1–S3—6288.

Adverse Event	n	% of Reports	Adverse Event	n	% of Reports
Angioedema	1108	24.1%	Stevens-Johnson syndrome	129	2.8%
Rash	847	18.5%	Anaphylactic shock	115	2.5%
Urticaria	480	10.5%	Fixed eruption	83	1.8%
Orbital or periorbital oedema	390	8.5%	Rash generalised	76	1.7%
Head, neck or respiratory tract oedema excluding orbital or periorbital area	319	7.0%	Lip swelling	75	1.6%
Hypersensitivity; Drug hypersensitivity	316	6.9%	Swelling face	72	1.6%
Eye swelling	263	5.7%	Rash erythematous	65	1.4%
Anaphylactic reaction	246	5.4%	Cough	65	1.4%
Erythema	173	3.8%	Blister	58	1.3%
Face oedema	163	3.6%	Maculo-papular rash	53	1.2%
Oedema in other than head or unspecified localisation	97	2.1%			

**Table 2:** Paracetamol Adverse Event reports grouped by target organ or system according to EudraVigilance “reaction groups” categories. The total sum of 5411 is greater than the total number of reports, because of the fact, that one report could be assigned to more than one reaction group.

Reaction Groups	Healthcare Professional	Non-Healthcare Professional	Not Specified/Missing	Total
Skin and subcutaneous tissue disorders	2657	493	16	3166
Eye disorders	532	116	2	650
Immune system disorders	559	159	4	722
General disorders and administration site conditions	230	0	0	230
Vascular disorders	178	37	1	216
Respiratory, thoracic and mediastinal disorders	172	53	0	225
Gastrointestinal disorders	136	29	2	167
Reproductive system and breast disorders	9	15	0	24
Ear and labyrinth disorders	4	4	0	8
Investigations	2	1	0	3

## Supplementary Materials

The following are available online at http://www.mdpi.com/2226-4787/7/1/12/s1, Table S1. Keywords selected from the particular “Reaction Groups” of EV database—part 1, Table S2. Keywords selected from the particular “Reaction Groups” of EV database—part 2, Table S3. Keywords selected from the particular “Reaction Groups” of EV database—part 3. Table S4. The hypersensitivity symptoms reported as AE with frequency <1.1%.
