# Supplementary material for: Hypersensitivity Reactions in Serious Adverse Events Reported for Paracetamol in the EudraVigilance Database, 2007–2018
Source: Pharmacy (Basel). 2019 Jan 17;7(1):12. doi: 10.3390/pharmacy7010012 (PMC6473647; doi:10.3390/pharmacy7010012)
Supplement: Supplementary file 1 [file pharmacy-07-00012-s001.pdf]

Table S1. Keywords selected from the particular "Reaction Groups" of EV database – part 1.

Ear and labyrinth disorders	Eye disorders	Gastrointestinal disorders	General disorders and administration site conditions	Immune system disorders
Ear swelling	Conjunctivitis allergic	Oral mucosa erosion*	Localised oedema[3]	Allergic oedema[3]
	Periorbital oedema[2]	Oral mucosal blistering*	Infusion site rash	Allergic reaction to excipient
	Eye oedema[2]	Oral mucosal exfoliation*	Injection site rash	Anaphylactic reaction**
	Eye swelling	Tongue blistering*	Oedema peripheral[3]	Anaphylactic shock**
	Eyelid oedema[2]	Stomatitis necrotising*	Peripheral swelling	Anaphylactoid reaction**
	Orbital oedema[2]	Swollen tongue	Swelling	Anaphylactoid shock**
		Tongue exfoliation*	Face oedema[1]	Drug hypersensitivity
		Tongue ulceration*	Generalised oedema[3]	Hypersensitivity
		Allergic colitis	Infusion site urticaria	Type I hypersensitivity
		Gastrointestinal oedema[3]	Injection site swelling	Type IV hypersensitivity reaction
		Lip oedema[1]	Infusion site oedema[3]	Type III immune complex mediated reaction
		Lip swelling	Oedema[3]	
		Mouth swelling	Oedema mucosal[3]	
		Oedema mouth[1]		
		Palatal oedema[1]		
		Palatal swelling		
		Tongue oedema[1]		

*keywords corresponding to prodromes or symptoms of Steven[illegible]hnson Syndrome/ Toxic Epidermal Necrolysis; ** keywords corresponding to diagnosis of anaphylaxis,[illegible]ywords corresponding to head, neck or respiratory tract oedema excluding orbital or periorbital area; [2] keywords corresponding to orbital or periorbital oedema; [3] keywords corresponding to oedema in other or unspecified localisation.

Table S2. Keywords selected from the particular "Reaction Groups" of EV database – part 2.

Investigations	Reproductive system and breast disorders	Respiratory, thoracic and mediastinal disorders		Vascular disorders
Eosinophil count increased	Genital erosion*	Catarrh	Bronchial obstruction	Flushing
Eosinophil percentage increased	Genital rash	Rhinorrhoea	Bronchial oedema[1]	Hot flush

Peak expiratory flow rate decreased	Genital swelling	Sinonasal obstruction	Bronchospasm	Shock
Allergy test positive	Genital ulceration	Sinus congestion	Cough	Shock symptom
	Oedema genital[3]	Sneezing	Epiglottic oedema[1]	
	Perineal ulceration	Pharyngeal erosion	Laryngeal oedema[1]	
	Scrotal oedema[3]	Status asthmaticus	Laryngeal stenosis	
	Scrotal swelling	Upper airway obstruction	Laryngospasm	
	Vulvar oedema[3]	Upper respiratory tract congestion	Laryngotracheal oedema[1]	
	Vulvar ulceration*	Allergic cough	Nasal oedema[1]	
	Vulvovaginal swelling	Asthmatic crisis	Oropharyngeal swelling	
	Vulvar erosion*	Bronchial disorder	Pharyngeal oedema[1]	
		Bronchial hyperreactivity	Respiratory tract oedema[1]	
			Tracheal oedema[1]	
			Wheezing	

* keywords corresponding to prodromes or symptoms of Stevens-Johnson Syndrome/ Toxic Epidermal Necrolysis; [1] keywords corresponding to head, neck or respiratory tract oedema excluding orbital or periorbital area; [2] keywords corresponding to orbital or periorbital oedema; [3] keywords corresponding to oedema in other or unspecified localisation.

Table S3. Keywords selected from the particular "Reaction Groups" of EV database – part 3.

**Skin and subcutaneous tissue disorders**		
Mucosal erosion	Vasculitic rash	Rash
Mucosal exfoliation	Acute cutaneous lupus erythematosus	Rash erythematous
Mucosal ulceration	Acute generalised exanthematous pustulosis	Rash follicular
Blister rupture	Angioedema	Rash generalised
Circumoral oedema[3]	Blister	Rash macular
Erythema multiforme	Dermatitis bullous	Rash maculo-papular
Fixed eruption	Dermatitis exfoliative	Rash morbilliform
Mucocutaneous rash	Dermatitis exfoliative generalised	Rash papular
Nikolsky's sign*	Drug eruption	Rash papulosquamous
Photosensitivity reaction	Drug reaction with eosinophilia and systemic symptoms	Rash pruritic
Skin erosion	Eczema vesicular	Rash rubelliform
Symmetrical drug-related intertriginous and flexural exanthema	Epidermal necrosis*	Rash scarlatiniform
Toxic epidermal necrolysis*	Erythema	Rash vesicular
Toxic skin eruption*	Exfoliative rash*	Skin exfoliation*

Urticaria	Generalised erythema	Skin oedema[3]
Urticaria papular	Hypersensitivity vasculitis	Skin swelling
Urticaria vesiculosa	Swelling face	Stevens-Johnson Syndrome*

*keywords corresponding to prodromes or symptoms of Stevens-Johnson Syndrome/ Toxic Epidermal Necrolysis; [1] keywords corresponding to head, neck or respiratory tract oedema excluding orbital or periorbital area; [2] keywords corresponding to orbital or periorbital oedema; [3] keywords corresponding to oedema in other or unspecified localisation.

Table S4. The hypersensitivity symptoms reported as AE with frequency <1.1%.

Adverse event	No of reports	Adverse event	No of reports
Erythema multiforme	46	Skin erosion	5
Rash pruritic	45	Epidermal necrosis	5
Shock	44	Oral mucosal blistering	4
Bronchospasm	43	Circumoral oedema	4
Acute generalised exanthematous pustulosis	42	Rash vesicular	4
Laryngeal oedema	41	Genital ulceration	3
Wheezing	39	Allergic cough	3
Swelling	37	Laryngospasm	3
Lip oedema	36	Nasal oedema	3
Drug eruption	34	Mucosal ulceration	3
Oedema peripheral	32	Photosensitivity reaction	3
Tongue oedema	31	Allergic oedema	2
Pharyngeal oedema	31	Anaphylactoid shock	2
Flushing	31	Type IV hypersensitivity reaction	2
Dermatitis bullous	30	Eosinophil count increased	2
Oedema	29	Genital erosion	2
Rash macular	28	Sinus congestion	2
Swollen tongue	27	Bronchial hyperreactivity	2
Hypersensitivity vasculitis	26	Tracheal oedema	2
Anaphylactoid reaction	25	Symmetrical drug-related intertriginous and flexural exanthema	2
Drug reaction with eosinophilia and systemic symptoms	25	Rash scarlatiniform	2
Peripheral swelling	24	Skin swelling	2
Oedema mucosal	19	Oral mucosal exfoliation	1
Rash papular	19	Tongue blistering	1

Skin exfoliation	18	Stomatitis necrotising	1
Type I hypersensitivity	17	Tongue ulceration	1
Vulvar erosion	16	Palatal swelling	1
Rhinorrhoea	16	Injection site swelling	1
Generalised erythema	15	Allergic reaction to excipient	1
Localised oedema	14	Type III immune complex mediated reaction	1
Hot flush	14	Peak expiratory flow rate decreased	1
Shock symptom	12	Genital rash	1
Dermatitis exfoliative	11	Perineal ulceration	1
Toxic skin eruption	11	Scrotal oedema	1
Ear swelling	10	Sneezing	9
Palatal oedema	9	Scrotal swelling	1
Asthmatic crisis	5	Catarrh	1
Respiratory tract oedema	8	Upper respiratory tract congestion	1
Nikolsky's sign	8	Epiglottic oedema	1
Mouth swelling	7	Oropharyngeal swelling	1
Oedema mouth	7	Mucosal exfoliation	1
Rash morbilliform	6	Blister rupture	1
Eye oedema	5	Dermatitis exfoliative generalised	1
Oral mucosa erosion	5	Urticaria vesiculosa	1
Mucosal erosion	5		
